# Interleukin-3 plays a vital role in hyperoxic acute lung injury in mice via mediating inflammation

**DOI:** 10.1186/s12890-018-0725-2

**Published:** 2018-10-30

**Authors:** Zhijian Huang, Wei Zhang, Jian Yang, Feiyu Sun, Hongwei Zhou

**Affiliations:** 1 Department of Emergency, Xia’men Traditional Chinese Medicine Hospital affiliated to Beijing University of Traditional Chinese Medicine, Xia’men, Fujian China; 2 Department of Respiratory, Jiangning Hospital affiliated to Nanjing Medical University, Nanjing, Jiangsu China; 3 Department of Intensive Care Unit, Xia’men Traditional Chinese Medicine Hospital affiliated to Beijing University of Traditional Chinese Medicine, No.1739 Xianyue Road, Xia’men, 361009 Fujian China

**Keywords:** Interleukin-3, Acute lung injury, Inflammation, Hyperoxia

## Abstract

**Background:**

Interleukin (IL)-3 amplifies inflammation. However, the effect of IL-3 in acute lung injury (ALI), an acute inflammatory disease, is unclear. The aim of this study was to test the hypothesis that IL-3 plays an important role in hyperoxia-induced ALI.

**Methods:**

Hyperoxic ALI was induced in wild-type (WT) and IL-3 gene disrupted (IL-3^−/−^) mice by exposure to 100% O_2_ for 72 h.

**Results:**

Hyperoxia increased IL-3 levels in plasma and lung tissues in WT mice. Pulmonary inflammation and edema were detected by histological assay in WT mice exposed to 100% O_2_ for 72 h. However, the hyperoxia-induced lung histological changes were improved in IL-3^−/−^ mice. The hyperoxia-induced elevation of neutrophils in bronchoalveolar lavage fluids and circulation were reduced in IL-3^−/−^ mice. Meanwhile, the levels of tumor necrosis factor-α and IL-6 were suppressed in IL-3^−/−^ mice compared with WT mice. Moreover, the hyperoxia-induced the activation of IκBα kinase (IKK) β, IκBα phosphorylation, and nuclear factor-κB translocation were inhibited in IL-3^−/−^ mice compared with WT mice.

**Conclusions:**

Our results suggest IL-3 is a potential therapeutic target for hyperoxia-induced ALI.

**Electronic supplementary material:**

The online version of this article (10.1186/s12890-018-0725-2) contains supplementary material, which is available to authorized users.

## Background

Acute respiratory distress syndrome (ARDS) remains a major challenge in intensive care medicine [1, 2]. Acute lung injury (ALI) is a mild form of ARDS. Inflammation is thought to contribute to the pathogenesis of ALI/ARDS [1–5], as ALI/ARDS is characterized by increased vascular permeability, extravasation of plasma, and neutrophil infiltration in the lung [1–4]. Thus, it is rational to explore anti-inflammatory therapies for this disorder [1, 5, 6]. However, it is bewildering that the results from clinical trials of novel anti-inflammatory strategies for ALI/ARDS have been disappointingly negative [1, 2, 4, 7]. These results reflect an incomplete understanding of ALI/ARDS pathogenesis. Therefore, the complicated cellular and molecular mechanisms contributing to the pathogenesis of ALI/ARDS needs to be further elucidated.

A recent study showed that interleukin (IL)-3 and its specific receptor α chain (IL-3Rα, also know as CD123) axis is responsible for cytokine storm during the pathogenesis of cecal ligation and puncture induced sepsis [8]. IL-3/CD123 axis is suggested as a potential therapeutic target for sepsis [8]. However, the underlying mechanism has not been adequately defined. Excessive cytokine-mediated inflammation plays a fundamental role in the pathogenesis of ALI/ARDS [9, 10], one of the most feared complications of sepsis [9, 11]. Nuclear factor (NF)-κB is known as a pivotal inducer of proinflammatory cytokines and highly activated in various inflammation-related diseases such as ALI [10, 12, 13]. A previous in vitro study reported IL-3 has potential effect on induction of IκBα kinase (IKK) β activation [14]. IKK plays a key role on regulation of NF-κB activation [15]. In the present study, we investigated the role of IL-3 in hyperoxia-induced ALI. Our findings may suggest new therapeutic target to prevent the onset of hyperoxic lung injury.

## Methods

### Animals

All animal experiments were performed in accordance with the National Institutes of Health guidelines for the use of experimental animals. All animal care and experimental procedures used in the present study were approved by the Ethics committee of Beijing University of Traditional Chinese Medicine. Healthy wild-type (WT) C57BL/6 mice (6–8 weeks old, body weight 16–20 g) were obtained from Beijing University of Traditional Chinese Medicine. IL-3 gene disrupted (IL-3^−/−^) mice on a C57BL/6 background were obtained from The Jackson Laboratory (Bar Harbor, ME). All of our current studies were performed using male mice. The animals were housed in individually ventilated cages under a 12 h light/dark cycle. Before the experiment, mice were habituated to the environment for at least 1 week. Standard chow and water were provided ad libitum. All procedures were performed as humanely as possible to minimize animal suffering.

### Experimental protocol

Mice were assigned to four groups (*n* = 8): sham+WT mice, sham+IL-3^−/−^ mice, hyperoxia+WT mice, and hyperoxia+IL-3^−/−^ mice. In brief, the mice were exposed to 100% O_2_ in a specially constructed plexiglas chamber to induce hyperoxic ALI [16]. Mice in sham group were exposed in room air. Seventy-two hours after hyperoxia or room air challenge, mice were sacrificed after anesthesia by pentobarbitone (50 mg/kg intraperitoneal injection) and were exsanguinated through the vena cava. Then, lung tissue sampling (for lung histology, and lung wet to dry weight ratio) was collected. Bronchoalveolar lavage fluids (BALF) and pulmonary tissue samples (for preparation of lung tissue homogenates) were collected in separate experiments.

### Lung histology

The lungs were fixed with formalin overnight. Five micrometer sections were deparaffinized. The sections were stained by haematoxylin/eosin (H&E). Lung injury scores were performed according to the following histological features: pulmonary edema, neutrophil infiltration, hyperemia, hemorrhage, and cellular hyperplasia. A score of 0 represented absent damage; l represented mild damage; 2 represented moderate damage; 3 represented severe damage [17].

### Lung water content

To quantify the magnitude of pulmonary edema, we evaluated the dry to wet (D/W) ratio of the lung. The left main bronchi were clamped, and the wet left lung was harvested. They were then placed in an oven for 48 h at 80 °C. Lung water content was calculated as (1-D/W) × 100%.

### Collection of BALF

The trachea was cannulated, and the lung was lavaged with 0.5 mL PBS for six times. The recovery rate of BALF was > 90% in all samples. Collected BALF was centrifuged at 1,200 rpm for 3 min. The supernatant was collected for further study.

### Enzyme linked immunosorbent assay (ELISA)

The concentrations of tumor necrosis factor (TNF)-α, IL-6, and IL-3 were measured by ELISA according to the manufacturer’s instructions (R&D Systems Inc., Minneapolis, MN, USA). The DNA-binding activity of NF-κB p65 was determined using an ELISA NF-κB p65 transcription factor assay kit according to the manufacturer’s instructions (Chemicon, Temecula, CA, USA).

### Western blotting analysis

Cytoplasmic and nuclear proteins were extracted from frozen lung tissue with the Nuclear/Cytosol Extraction kit (BioVision, Inc., Mountain View, CA, USA) according to the manufacturer’s instructions. Protein concentrations were determined using the bicinchoninic acid protein assay (Pierce, Rockford, IL, USA). 50 μg of total protein were subjected to SDS-PAGE and transferred onto PVDF membranes. Membranes were blocked with 5% non-fat milk at room temperature for 3 h, incubated with primary antibodies (Anti-NF-κB p65 (sc-7151) and phosphorylated IκBα antibodies (sc-7977), diluted 1:500, Santa Cruz Biotechnology, Santa Cruz, CA, USA; Anti-phosphorylated IKKα/IKKβ antibody (2078), diluted 1:500, Cell Signaling, Boston, MA, USA; Anti-IL-3 antibody (AF-403-NA), diluted 1:200, R&D Systems Inc., Minneapolis, MN, USA; Anti-CD123 antibody (106002), diluted 1:200, Biolegend, San Diego, CA, USA) at 4 °C overnight. β-actin (3700) and Lamin B (12586) (Cell Signaling, Boston, MA, USA) were used as an internal control for cytoplasmic and nuclear protein, respectively. On the next day, membranes were incubated with HRP-conjugated secondary antibodies (Cell Signaling, Boston, MA, USA) at 37 °C for 1 h. Protein bands on the membrane were visualized with ECL Kit (Biovision, Milpitas, CA, USA) using FluorChem FC3 system (ProteinSimple, San Jose, CA, USA). Results were presented as densitometric ratio between the protein of interest and the loading control.

### Survival study

The survival rate was observed at 24-h intervals. Observation was continued 72 h.

### Statistical analysis

All data were analyzed with GraphPad Prism 6.0 (GraphPad Software, CA, USA) and were presented as mean ± SEM. Two-way ANOVA with Bonferroni’s multiple-comparisons test was used for multiple group analysis. Histopathologic scores were compared using the Mann-Whitney *U* test. The survival rate was estimated by the Kaplan-Meier method and compared by log-rank test. *P* < 0.05 was accepted as statistically significant.

## Results

### Effect of hyperoxia on IL-3 and IL-3Rα

**Fig. 1:**
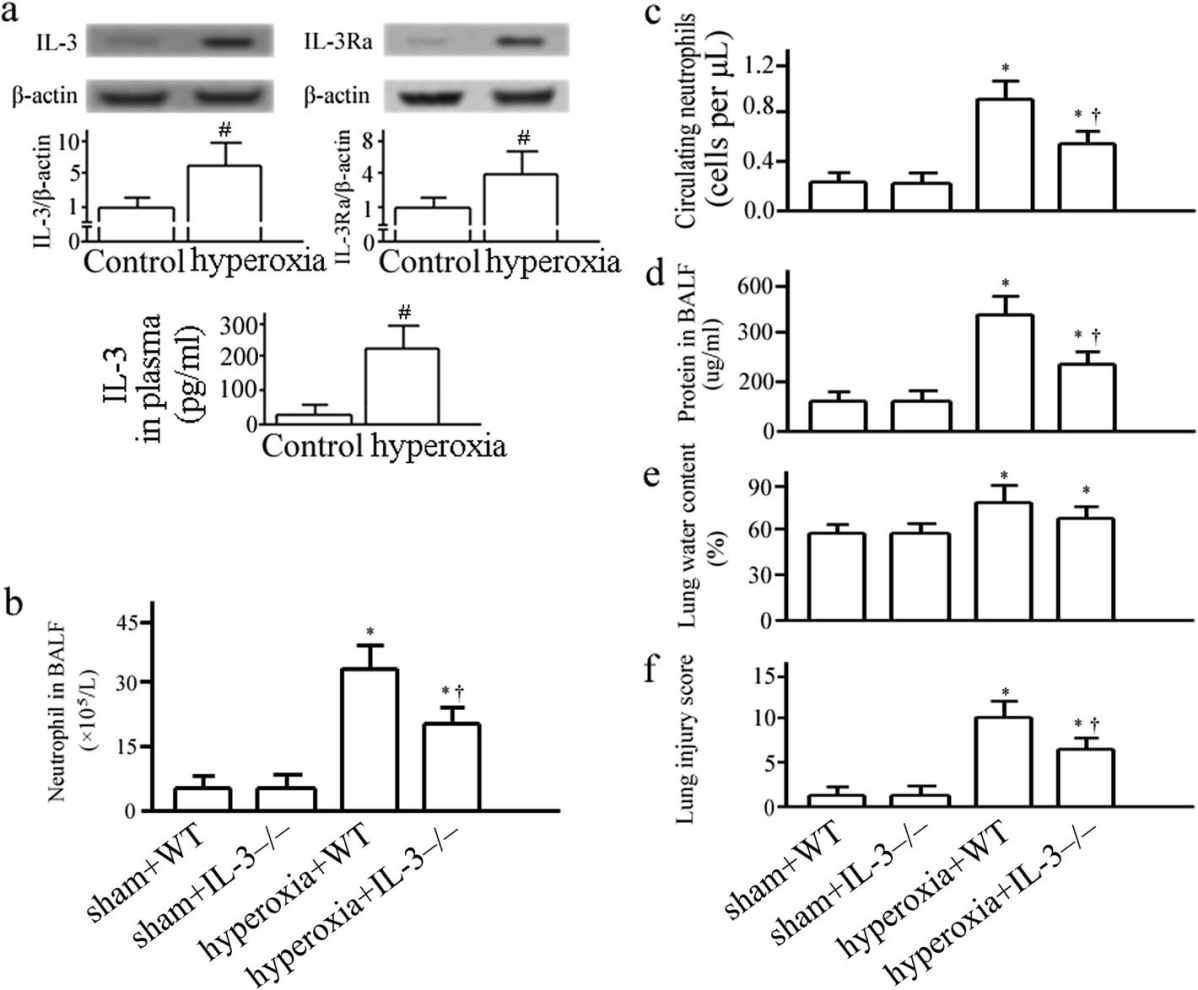
Effect of hyperoxia on interleukin (IL)-3 and IL-3-specific receptor α chain (IL-3Rα). C57BL/6 mice were challenged with room air (control) or hyperoxia for 72 h and pulmonary protein of IL-3 and IL-3Rα were assessed by western blotting, and IL-3 levels in plasma was measured by enzyme linked immunosorbent assay (ELISA) (**a**). Neutrophils in bronchoalveolar lavage fluids (BALF) (**b**) and circulation (**c**), protein in BALF (**d**), lung edema (**e**), and lung injury score (**f**) were assessed 72 h after hyperoxia or room air challenge. Data represent assessments in a minimum of *n* = 5 mice. ^#^*P* < 0.05 vs. control; ^*^P < 0.05 vs. sham+wild type (WT) group; ^†^ *P* < 0.05 vs. hyperoxia +WT group. IL-3^−/−^, IL-3 gene disrupted mice

The levels of IL-3 and IL-3Rα in the lung were detected by Western Blotting in additional groups of animals (Fig. 1a and Additional file 1: Figure S1). Hyperoxia caused significant increase of IL-3 in the lung (Fig. 1a). It was simultaneously associated with an increase in expression of IL-3Rα in the lung 72 h after hyperoxia challenge (Fig. 1a). Moreover, the concentration of IL-3 in plasma was significantly elevated in hyperoxia exposure group compared with sham (Fig. 1a).

### Effects of IL-3 on lung injury and mortality in hyperoxia-induced ALI

**Fig. 2:**
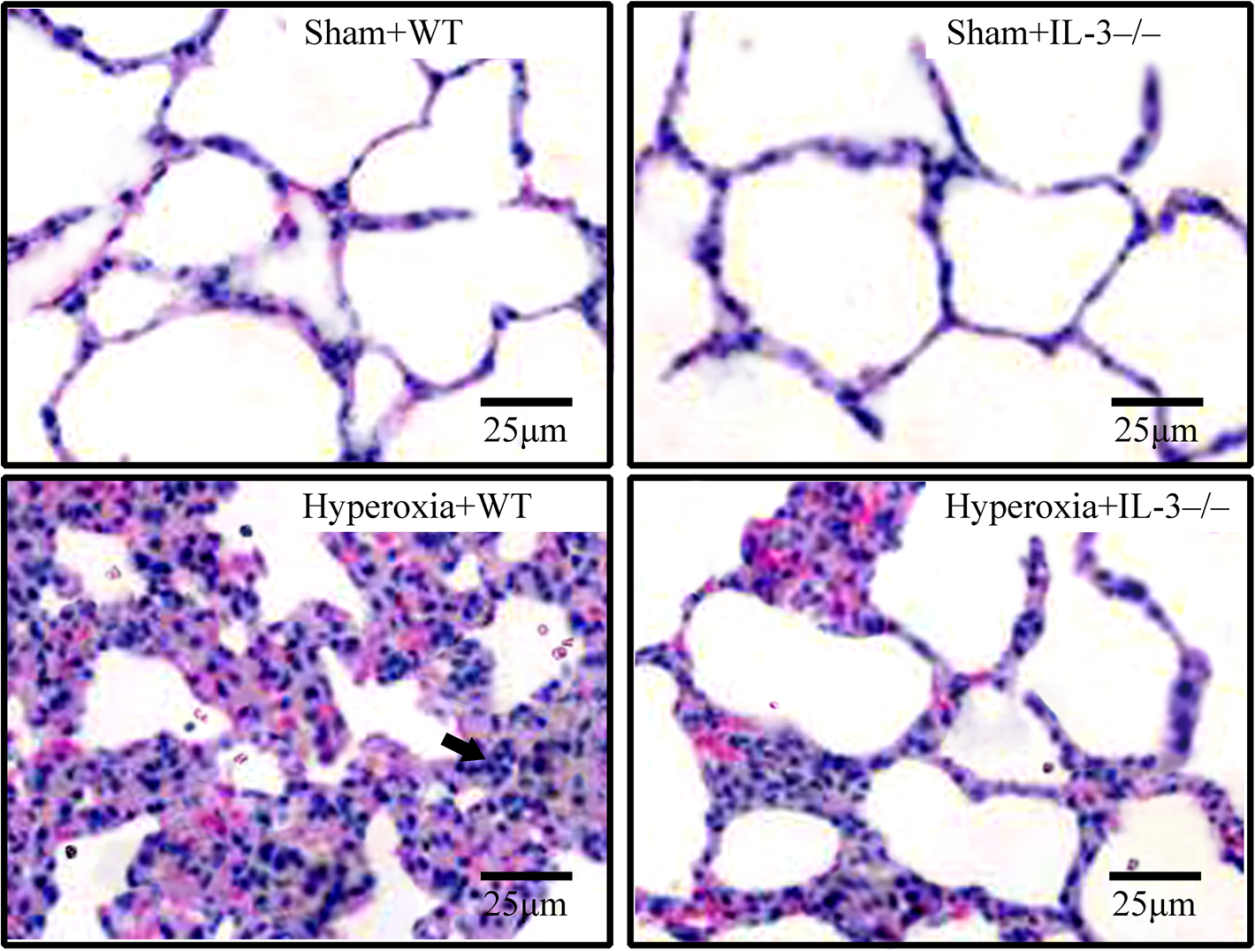
Lung histological features were assessed 72 h after hyperoxia or room air challenge by light microscopy, hematoxylin and eosin stain in wild-type mice (WT) and interleukin-3 gene disrupted mice (IL-3^−/−^). Original magnification, × 400

Hyperoxia caused significant neutrophil infiltration, alveolar capillary protein leak, and lung edema after 72 h, which could be dampened in IL-3^−/−^ mice (Fig. 1b, c, d, and e). When IL-3^−/−^ mice were treated with hyperoxia, the lung histological changes were reduced compared with WT mice (Fig.1f; Fig. 2). All mice died within 3 days in hyperoxia+WT group. In contrast, mice were more resistant to hyperoxia in IL-3^−/−^ mice. A total of 50% of the mice in the WT mice treated hyperoxia group died within 24 h, and an additional 50% died within 72 h, while 60% of the mice in the IL-3^−/−^ mice treated hyperoxia group survived.

### Effects of IL-3 on proinflammatory mediators in hyperoxia-induced ALI

**Fig. 3:**
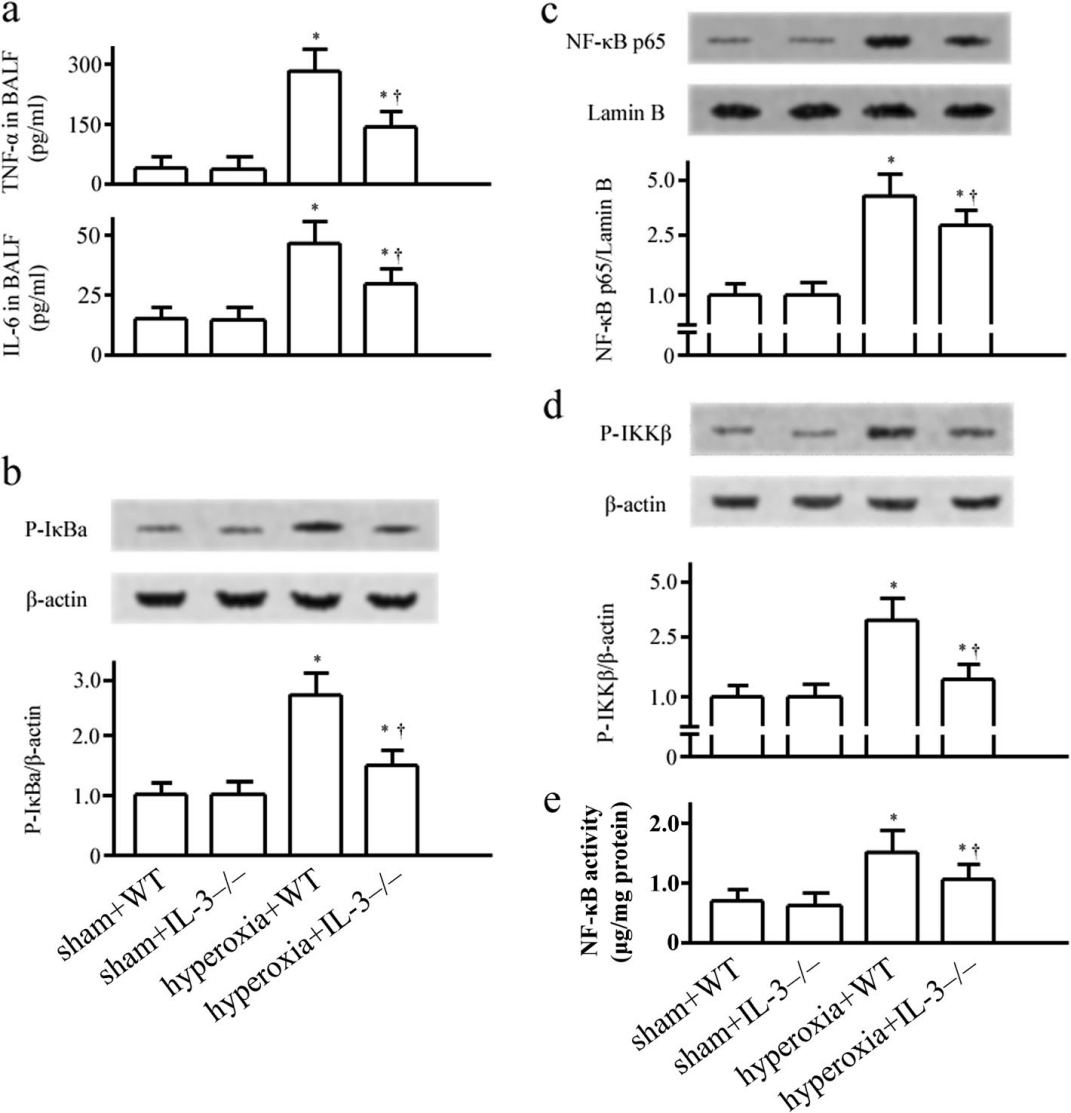
Effect of IL-3 on proinflammatory mediators and nuclear factor (NF)-κB activation in mice. Tumor necrosis factor (TNF)-α and interleukin (IL)-6 (**a**) in bronchoalveolar lavage fluids (BALF), phosphorylated (p)-IκBα in the cytoplasm (**b**), nuclear factor (NF)-κB p65 in the nucleus (**c**), p-IκBα kinase (IKK) β (**d**), and NF-κB activity (**e**) in lung tissues were assessed 72 hours after hyperoxia or room air challenge by enzyme linked immunosorbent assay (ELISA) or western blotting. Data represent assessments in a minimum of n = 5 mice. *P <0.05 vs. sham+wild type (WT) group; † *P* <0.05 vs. hyperoxia+WT group. IL-3–/–, IL-3 gene disrupted mice

As proinflammatory cytokines have a key role in ALI [10, 12], the IL-6 and TNF-α in BALF were studied in our study. Seventy-two hours after hyperoxia challenge, there was a significant reduction in TNF-α and IL-6 concentrations in IL-3^−/−^ mice versus WT mice (Fig. 3a).

### Effects of IL-3 on IKK/NF-κB pathway

Hyperoxia exposure induced IκB phosphorylation and NF-κB p65 nuclear translocation in the lung (Fig. 3b, and c). NF-κB p65 levels in the nucleus and phosphorylated IκBα levels in the cytoplasm were reduced in IL-3^−/−^ mice (Fig. 3b, and c). The hyperoxia-induced IKKβ activation was dampened in IL-3^−/−^ mice (Fig. 3d). Hyperoxia exposure induced activation of NF-κB was reduced in IL-3^−/−^ mice (Fig. 3e).

## Discussion

IL-3 was first reported to be a potential new therapeutic target for sepsis in 2015 [8]. However, the effect of IL-3 in ALI has been generally understudied. Our results showed that IL-3 gene deleted mice have improved lung inflammation and edema in hyperoxia-induced ALI. Moreover, hyperoxia-induced the activation of IKK/NF-κB signaling pathways and upregulation of proinflammatory mediators were reduced in IL-3^−/−^ mice.

Lines of evidence have shown that IL-3 is released by activated Th2 lymphocytes which play crucial roles in allergic disorders [8, 18]. IL-3 is also known as multi-potential colony-stimulating factor which stimulating proliferation of pluripotent hematopoietic stem cells and progenitor cells [19–21]. A recent study suggests that IL-3 plays a vital role in sepsis [8], an infectious disorder [9, 11]. IL-3 plays its effect via combining with its receptor. The IL-3 receptor is a heterodimer which composed of one α chain and oneβchain [19, 22]. The α chain is IL-3 specific receptor also known as CD123. IL-3Rα is expressed in hematopoietic stem and progenitor cells, dendritic progenitors, and macrophage [8, 19]. These cells were infiltrated in the lung in the pathogenesis of ALI. In the present study, a significant increase in IL-3 and IL-3Rα levels in the lung homogenates 72 h after hyperoxia stimulation was detected by Western Blotting.

Over released proinflammatory mediators are crucial to the initiation of inflammatory tissue injury [9]. The influence of IL-3 and it receptor on proinflammatory mediators has been reported [8, 14, 23, 24]. A previous in vitro study suggests a potential posttranscriptional regulation effect of IL-3 on TNF-α via a p38-mitogen-activated protein kinase and silent information regulator type-2-dependent manner [23]. The limiting role of anti-CD123 in cytokine secretion has been recognized previously [8]. Our data showed that TNF-α and IL-6 were reduced in IL-3 gene deleted mice compared with WT mice at 72 h after hyperoxia stimulation. However, little is known regarding the underlying mechanisms. IKK/NF-κB pathway is one critical transcriptional mechanism required for maximal expression of many cytokines involved in the pathogenesis of ALI [10, 12]. A previous in vitro study reported IL-3 induces the activation of IKKβ in mast cell via a Src family kinase and Ca^2+^ dependent manner [14]. Activation of IKK induces phosphorylation and degradation of IκB, leading to the nuclear translocation of NF-κB and transcriptional activation [15]. Our results showed that the IL-3 levels in the lung and plasma were significantly elevated in hyperoxia exposure group compared with sham. Meanwhile, the IKK/NF-κB pathway was activated by hyperoxia exposure. However, when the IL-3 gene was deleted in mice, the hyperoxia-induced the activation of IKK/NF-κB pathway and cytokine productions were dampened. Our results suggest that IL-3 is associated with the activation of IKK/NF-κB pathway. However, the precise mechanism responsible for the effect of IL-3 on the IKK/NF-κB pathway and cytokine productions warrants further investigation.

Overall, our data suggest that IL-3 and its receptor IL-3Rα are induced during hyperoxia-induced lung injury, and thus further mediate inflammation via IKK/NF-κB axis to promote proinflammatory cytokine production and elevate inflammation.

## Conclusions

In summary, our findings show that IL-3 and IL-3Rα are stimulated in mice hyperoxia-induced ALI. Deletion of IL-3 reduced hyperoxia-induced ALI. Our results suggest IL-3 is a potential therapeutic target for hyperoxia-induced ALI.

## Additional file

Additional file 1: **Figure S1.** Expression of interleukin (IL)-3 in the lung in wild-type (WT) and IL-3 gene disrupted (IL-3^−/−^) mice was detected by western blot. (TIF 583 kb)

## Abbreviations

ALI: Acute lung injury
ANOVA: Analysis of variance
ARDS: Acute respiratory distress syndrome
BALF: Bronchoalveolar lavage fluid
ELISA: Enzyme-linked immunosorbent assay
IKK: IκBα kinase
IL-3: Interleukin-3
IL-3Rα: Interleukin-3 specific receptor α chain
ROS: Reactive oxygen species
TNF-α: Tumor necrosis factor
W/D: Lung wet/dry weight ratio
WT: Wild-type
