# Supplementary figures and images for: Interleukin-3 plays a vital role in hyperoxic acute lung injury in mice via mediating inflammation
Source: BMC Pulm Med. 2018 Oct 30;18:164. doi: 10.1186/s12890-018-0725-2 (PMC6206653; doi:10.1186/s12890-018-0725-2)

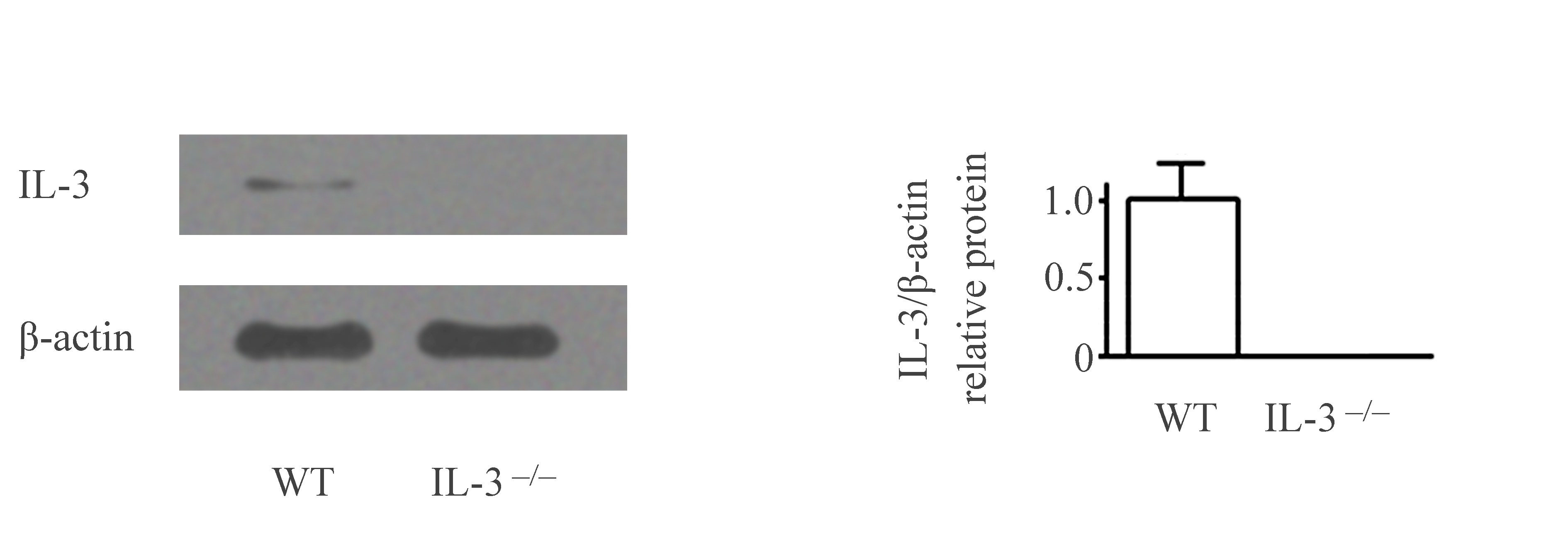

Supplement: Supplementary file 1 — Figure S1. Expression of interleukin (IL)-3 in the lung in wild-type (WT) and IL-3 gene disrupted (IL-3−/−) mice was detected by western blot. (TIF 583 kb) [file 12890_2018_725_MOESM1_ESM.tif]
